# Integrating patient management, reflective practice, and ethical decision-making in an emergency medicine intern boot camp

**DOI:** 10.1186/s12909-021-02970-8

**Published:** 2021-10-22

**Authors:** Serpil Yaylaci, Yesim Isil Ulman, Kevser Vatansever, Gamze Senyurek, Suha Turkmen, Hasan Aldinc, Cem Gun

**Affiliations:** 1 Department of Emergency Medicine, Acibadem Mehmet Ali Aydinlar University, School of Medicine, Istanbul, Turkey; 2 Department of History of Medicine and Ethics, Acibadem Mehmet Ali Aydinlar University, School of Medicine, Istanbul, Turkey; 3 Department of Medical Education, Ege University, School of Medicine, İzmir, Turkey; 4 Acibadem Mehmet Ali Aydinlar University, Graduate School of Health Sciences, Bioethics Master Program, Istanbul, Turkey

**Keywords:** Clinical decision-making, Emergency medicine, Ethical reasoning, Ethics, Internship, Reflective practice

## Abstract

**Background:**

Integration of clinical skills, ethical decision-making, and reflection skills have emerged as cornerstones of clinical teaching in medical schools. This study aimed to detect whether a multimodal learning environment approach consisting of lectures, a drill, post-drill video debriefing, and written reflection in an emergency medicine rotation boot camp improves interns’ patient management skills, ethical decision-making, and reflection skills.

**Methods:**

A multimodal learning environment was created by the collaboration of emergency medicine, ethics, and medical education specialists. Multiple educational techniques involving lectures, case discussions, and role-playing a crisis scenario were applied. Pre-test and post-test, debriefing on performances on video records, video-recorded performance assessment, and reflective essays about their own and group’s performances were used to assess various aspects of the student performances. Additionally, a meeting was organized with the presence of the authors to create qualitative data obtained through the program evaluation meeting conducted on three themes: influences of teaching methods, students’ performances, and common achievements and mistakes of students.

**Results:**

133 students participated. Post-test multiple-choice question (MCQ) test scores were slightly higher than pre-test. A low and medium correlation was detected among pre-test and post-test patient management problem (PMP) and reflection scores, which was more prominent for female students. Multiple linear regression showed that pre-test and post-test PMP scores significantly contributed to reflection scores. These results might support that better patient management predicts more robust reflective practice. Teachers observed that students appreciated being inspired by well-performing peers, particularly noting the empathic needs of patients, companions, and other health professionals. However, students overlooked summoning forensic or social services and were inhibited by the pressure of the contextual traits of the drill.

**Conclusion:**

The multimodal learning environment created by multidisciplinary collaboration contributed to the improvement of components of situational awareness of the interns: patient management skills, ethical decision-making, and reflective practice. During this research, we created a toolbox better to capture the richness and diversity of student interactions. Considering the scarcity of context-specific assessment methods and widespread use of MCQs or generic scales for higher-order thinking skills in medicine, this study might be regarded as a step forward in that context.

**Supplementary Information:**

The online version contains supplementary material available at 10.1186/s12909-021-02970-8.

## Introduction

Emergency medicine operates against time pressures in the care of patients in life-threatening situations and entails numerous ethical dilemmas. Although fundamental ethical obligations such as informed consent and confidentiality might sometimes not be fully met for patients in emergency care, respect for patient autonomy, beneficence, nonmaleficence, privacy and confidentiality, and fairness must be balanced with optimal care [1, 2] in line with sound ethical decision-making.

Reflection is an important step in ethical reasoning and decision-making and involves the following steps: analyzing the underlying dilemma, realizing duties and responsibilities in terms of conflicting values, presenting a solution in the light of ethical principles, and decision testing [3, 4]. Strategies to enhance the learning of clinical reasoning include both internally focused reflection and external reflective verbal or written articulation [5]. In medical education, reflection mostly occurs after the event (reflection-on-action). Written exercises, or recently digital records, are suggested as guided writing of reflection on the action and are advantageous because of the use of a structured design that is a critical aspect for enhancing reflection [5, 6].

Tsai and Harasym noted the importance of considering medical indications, quality of life, the patient’s preferences, and the current context in ethical reasoning [7]. This results in transformative learning, which was described by Mezirow as self-reviewing in the presence of a disorienting dilemma, performing the premise reflection, recognizing the change in others, formulation of an action plan, acquisition of the knowledge and skills required to execute the plan, competency, and self-confidence improvement for new roles, and re-adaptation to life with new perspectives [8]. Effective reflection requires time, effort, willingness to question actions, beliefs, values, and to search for other viewpoints. This approach moves beyond the single loop of solely seeking an alternative plan or the double loop to find out the reasons for the outcome, and proceeds to a triple loop of transformation that necessitates questioning the underlying conceptual frameworks [6]. The most important point is to provide students with the opportunities to reflect and reveal their deepest thoughts and views in the presence of a role model in a safe, simulated environment [9, 10].

Debriefing is a purposeful, structured reflection technique that involves reflecting on the experienced event, discussing experiences with others, and learning and modifying behaviors [10]. Evaluation of reflection is essential since it motivates learning and especially shared reflection is better than individual and self-assessment [6]. Reflective writing is also reported to produce a profound improvement in students’ professional affective skills [11], and rubric scales are proposed as an appropriate method for analyzing the level of reflection [12].

In the last decades, integration of clinical skills, ethical decision-making, and reflection skills have emerged as cornerstones of clinical teaching in medical schools, but all are seldom linked in curricula [13]. The innovative program of Brunger et al. [13] in the first year of Faculty of Medicine of Memorial University that aimed to integrate critical self-reflection, reflection on the political, economic, and cultural contexts shaping health and health care, and moral decision-making, could be looked into as one of the few examples in medical education. As well as medical education, there are several studies in health professionals education, i.e. nursing and physical therapy, discussing the integration of clinical reasoning with ethical reasoning or with reflection [14–19]. However, there seems to be a gap in the literature on health professionals’ education related to the teaching of clinical reasoning/decision-making, reflection, and ethical reasoning/decision-making in a manner that integrates all three aspects.

It was noticed that in the literature, assessment of ethics skills was solely based on pre- post-tests, multiple-choice questions (MCQs), or generic test batteries such as Defining Issues Test or Moral Judgment Tests [20–24]. Additionally, there are also studies reporting the use of performance tests with virtual or standardized patients [25–27].

At the Acibadem Mehmet Ali Aydinlar University School of Medicine (SoM), medical ethics, bioethics, and professionalism are integrated into the curriculum longitudinally in phase one (years 1–3). In phase 2, (years 4–5) “Ethics Rounds” are practiced within the clinical clerkships [28]. The aim of these is to integrate previously learned ethical issues into clinical training [29, 30]. In phase 3 (year 6), integration of both clinical and ethical decision-making with reflection skills constitutes an example of a spiral and vertically integrated program for teaching those higher-order thinking skills, which are crucial for the nurturing of virtuous physicians [31].

During the emergency medicine internship in phase 3, a novel module has been implemented to blend clinical reasoning, ethical decision-making, and reflection in an emergency care drill during the rotation before emergency department attachment. The novel module was included in the abdominal pain day of a 5-day long boot camp that focused on diagnosing and managing different life-threatening situations. Each day, situations such as altered mental status, chest pain and dyspnea, multiple traumas, and a busy emergency department were taught. In addition, high-fidelity simulators were utilized, the faculty and intern doctors were included in role-playing scenarios, and the interns were given a chance for reflection throughout [32]. The learning outcomes of this boot camp were improving difficult situation management, reflection and communication skills, and patient management in an integrated, interactive, feedback-given environment.

This study aimed to examine the relationships between patient management skills, ethical decision-making, and reflective practice to introduce the multimodal learning environment designed for interns at the Acibadem Mehmet Ali Aydinlar University SoM to improve clinical management, ethical reasoning, and reflection skills. The environment was introduced in the five-day-long boot camp of the emergency medicine rotation. Lastly, it was aimed to present the study results obtained through the quantitative assessment methods and the qualitative data obtained through a program evaluation meeting.

## Methods

This study was approved by the Acibadem Mehmet Ali Aydinlar University Research Ethics Committee (ATADEK) with the decision number: 2018–2/68. The written informed consent was obtained from the participants before the practice. All methods were performed in accordance with the relevant guidelines and regulations.

### Technical information

#### Hypothesis

In an emergency medicine rotation intern boot camp, does a multimodal learning environment approach consisting of lectures, a drill, post-drill video debriefing, and written reflection improve the patient management skills, ethical decision-making, and reflective practice of interns?

### Study design

A multimodal learning environment was created by the multidisciplinary collaboration of emergency medicine, ethics, and medical education specialists. This study was created as a multi-method design consisting of quantitative student assessment methods, and also presents the qualitative data obtained through the program evaluation meeting conducted with the presence of the authors SY, YIU, GS, KV.

Multiple educational techniques involving lectures, case discussions, and role-playing a crisis scenario were applied. Pre-test and post-test, debriefing on performances on video records, video-recorded performance assessment (VPA), and reflective essays about their own and group’s performances were used for the assessment of various aspects of the student performances. Additionally, a meeting was organized with the presence of the authors SY, YIU, KV, and GS to evaluate the program. During the meeting, observations, and opinions of authors as teachers were obtained on three themes: (1) influences of teaching methods, (2) students’ performances, and (3) common achievements and mistakes of students.

The study group consisted of interns from the Acibadem Mehmet Ali Aydinlar University SoM in their emergency medicine rotations. The research was conducted over two years (2018–2019) with eight consecutive groups undergoing “Abdominal Pain Day” of the five-day-long boot camps before the beginning of the emergency medicine rotation (Table 1).

**Table 1:** The emergency medicine internship rotation boot camp abdominal pain management day program

Program Information	Duration (minutes)
Abdominal Pain Management Lecture	60
Three Abdominal Pain Cases per each Group	90
Pre-test	15
Emergency Care and Ethics Lecture	30
Explanations about the Drill	10
Lunch	60
Drill	60
Post-test	15
Video Debriefing	60
Written Essay Reflection	10

### Learning outcomes

The program aimed to assist the interns in preparation for clinical and ethical decision-making in their prospective professional lives. Targeted learning outcomes include:

1. Applying basic principles of clinical management of life-threatening conditions
2. Acknowledging the rules and principles of ethics in emergency health care services
3. Practicing informed consent with emergency patients
4. Articulating the main attributes of ethical decision-making
5. Analyzing the components of difficult situation management
6. Experimenting with empathic, effective, and participative communication with patients and companions

The interns were given a one-hour lecture and a discussion session on the management of abdominal pain in the emergency department. Following this, a pre-test was administered. The goals and expectations concerning the drill were explained and the students were asked to select opposite gender roles during role-playing to experience empathy with the opposite gender.

A post-test that was identical to the pre-test was applied. Seven MCQs for assessing remembering and comprehending and three patient management problems (PMPs) for assessing the cognitive skills of applying and analyzing were used (Additional file 1). After the post-test, in a video debriefing session, students evaluated and reflected on the role-play, discussed their own experiences, and described what had been learned and what else was needed for better performance. Assessment tools consisted of rubric forms; one was used for students’ reflective essays and the other was used for VPA. The reflective essays were also used as a tool for self-directed learning by reviewing these with students on the third week of the rotation and by asking them to assess their progress and their plans to improve further.

As for qualitative data, observations and opinions of the authors SY, YIU, KV, and GS that were obtained in the program evaluation meeting were presented. The meeting was focused on the observations and opinions on (1) evaluating the influences of teaching content and methods, (2) students’ performances, and (3) common achievements, mistakes, and stereotypes of the interns. Meeting notes were discussed in the presence of all and decided to be reported in this manuscript as qualitative data.

The drill scenario consisted of five roles: physician, nurse, patient, the patient’s mother, and the patient’s brother. These were designed according to a hidden curriculum aspect consisting of one adult and one minor patient, both of whom were hemodynamically unstable due to a rupture of ectopic pregnancy as a result of an extramarital relationship. All roles were played by the interns. The video recording was then watched with the interns, and a debriefing was given. The drill was concluded with the reflective essays from the interns.

The footage recorded during the drill was assessed by two teachers (one ethicist and one emergency medicine physician) and was scored in line with the VPA rubric form. If the scores of two teachers were inconsistent, they negotiated and settled on an agreed score. The group performance results were adopted as the individual interns’ VPA scores. The VPA topics on the rubric form are presented in Tables 2 and 3 below:

**Table 2:** Video performance assessment topics and scoring

Topics	Points
**Clinical Management of Life-Threatening Condition**	**10**
Was the patient’s medical situation evaluated appropriately?	2
Were the necessary tests ordered?	2
Was the patient’s problem understood from a clinical perspective?	2
Was the patient diagnosed correctly?	2
Were decisions made regarding the follow-up of the patient?	2
**Ethical Decision-Making**	**10**
Was the patient’s ability to decide evaluated? Was the needed consultation requested?	2
Were optimal patient and professional privacy achieved during the physical examination?	2
Was informed consent obtained before an intervention?	2
Were optimal patient and professional privacy achieved in general?	2
Were the patient’s companions given appropriate information without compromising the patient’s privacy?	2
**Difficult Situation Management**	**4**
Were the insistent and inpatient behaviors appropriately managed?	1
Was an appropriate therapeutic environment achieved by excluding the patient’s companions?	2
Was a task-sharing approach adopted to control the companions?	1
**Communication Skills**	**16**
Was the patient greeted appropriately?	1
Were the patient’s concerns relieved?	2
Was the physical examination carried out with an adequate attitude?	2
Were the patient’s companions calmed down with a soothing and informative attitude?	2
Was the patient spoken with privately to understand his or her problem?	2
Was the patient informed in a comprehensible manner?	2
Did the doctor check the patient’s understanding of the situation?	1
Was the patient given enough time to decide?	2
Were the patient’s questions answered adequately?	2

**Table 3:** Reflective essay rubric form and scoring

Reflective Essay Rubric Form	
**Event Description**	0 points, superficial evaluation: 1-point, partial reflection: 3 points, in-depth reflection: 5 points
What happened in the drill?	
What surprised you?	
**Feelings and Thoughts**	
What were your feelings?	
**Evaluation and Analysis**	
What did you do well?	
What would you do better?	
**Conclusion**	
Which aspects would you wish to improve?	
**Action Plan**	
What is your plan for improving your performance in a similar situation?	

### Statistical analysis

Descriptive statistics were presented using mean, standard deviation, median, minimum, maximum. Paired Samples t-test was used to compare two normally distributed dependent groups.

Spearman’s rho correlation analysis was used to analyze relationships between two non-normally distributed variables. Multiple linear regression analysis was used to predict interns’ reflective essay scores. The data categories from 133 interns were as follows: reflective essay scores, gender, VPA scores, pre-test and post-test MCQs and PMPs. The backward variable selection method was used.

Statistical significance was accepted when two-sided *p* values were lower than 0.05.

Statistical analysis was performed using MedCalc Statistical Software version 12.7.7.

## Results

The research sample consisted of all 133 interns of the Acibadem Mehmet Ali Aydinlar University SoM attending emergency medicine internship in eight groups consisting of 12–21 students (Mean: 16.63). Seventy-seven (57.9%) students were women and 56 (42.1%) were men. Students’ mean age was 24.1 ± 1.26 years (Median: 24; Interquartile Range: 22–31).

Table 4 shows the average scores of students for all assessment methods. Detailed research data is available in Additional file 2.

**Table 4:** Descriptive statistics of assessment scores

Assessment Tools	Mean + Standard Deviation	Median (Minimum-Maximum)	Significance
Pre-test MCQs	8.51 ± 1.20	9 (5–10)	p: 0.010
Post-test MCQs	8.77 ± 1.12	9 (5–10)	
Pre-test PMPs	7.28 ± 2.28	9 (0–9)	p: 0.261
Post-test PMPs	6.98 ± 2.65	9 (0–9)	
VPA	28.86 ± 7.42	29.5 (0–40)	
*Clinical management of life-threatening condition*	7.15 ± 1.56	7 (4–10)	
*Ethical decision-making*	7.12 ± 1.96	7 (3.5–10)	
*Difficult situation management*	2.79 ± 0.85	3 (1–4)	
*Communication skills*	12.01 ± 3.38	12 (2–16)	
Reflective essay	18.76 ± 5.71	18 (6–30)	
Total score (post-test + VPA + reflective essay)	63.40 ± 11.02	64 (25–86)	

*MCQ* Multiple-Choice Question, *PMP* Patient Management Problem, *VPA* Video-recorded Performance Assessment

The total post-test MCQ score was slightly higher than the pre-test MCQs (p: 0.010). The difference in the post-test PMPs was not found to have a statistically significant difference with the pre-test PMPs. The mean VPA scores were 28.86 out of 40, the mean reflective writing score was 18.76 out of 30, and the mean total post-test score was 63.40 out of 86.

Correlation coefficients of total and all reflection questions’ scores, and VPA scores with pre-test and post-test MCQ and PMP are presented in Table 5.

**Table 5:** Correlation of pre-test and post-test multiple-choice questions and patient management problems with video performance assessment and reflection essay scores

Groups	Reflection Question 1	Reflection Question 2	Reflection Question 3	Reflection Question 4	Reflection Question 5	Reflection Question 6	Total Reflective Essay	VPA Score
**Total**								
Pre-test MCQ	0.072	−0.064	0.124	−0.031	0.017	0.068	0.049	0.136
Post-test MCQ	0.079	−0.030	0.058	0.049	0.000	0.157	0.079	0.272**
Pre-test PMP	0.215*	0.227**	0.171*	0.279**	0.195*	0.192*	0.265**	0.049
Post-test PMP	0.185*	0.182**	0.055	0.324**	0.147	0.151	0.220*	0.070
**Male**								
Pre-test MCQ	0.010	−0.028	0.186	−0.066	−0.148	−0.025	0.019	0.172
Post-test MCQ	−0.018	0.047	0.072	0.207	−0.038	0.084	0.079	0.154
Pre-test PMP	0.168	0.159	0.232	0.309*	−0.064	0.107	0.177	0.155
Post-test PMP	0.062	0.192	0.113	0.388**	0.115	0.186	0.234	0.063
**Female**								
Pre-test MCQ	0.122	−0.092	0.098	0.016	0.122	0.152	0.101	0.110
Post-test MCQ	0.172	−0.090	0.063	−0.063	0.026	0.245*	0.103	0.399**
Pre-test PMP	0.253**	0.284*	0.141	0.277*	0.357**	0.262*	0.357**	−0,004
Post-test PMP	0.264*	0.177	0.021	0.293**	0.168	0.138	0.220	0,108

*VPA* Video-recorded Performance Assessment, *MCQ* Multiple-Choice Questions, *PMP* Patient Management Problems) Spearman’s rho,p * < 0.05, ** < 0.001.

Total scores of pre-test and post-test MCQ were found to be moderately correlated (0.46). Pre-test and post-test PMP scores were also found to have a moderate correlation (0.404). A weak correlation between pre-test MCQ and VPA was detected (0.272). For female students, post-test MCQ scores showed a moderate correlation with VPA scores (0.399). Reflection essay scores had no correlation with the pre-test and post-test MCQs, except low correlation of reflection question 6 for male students (0.345). There was a weak correlation of all reflection essay questions and total scores with pre-test PMP and post-test PMP scores, changing from 0.182 to 0.279 (Table 5). This correlation was more prominent among female interns for pre-test PMP scores, changing from 0.253 to 0.357.

The multiple linear regression to predict reflection scores from gender, pre-test, and post-test MCQ, PMP, and VPA scores are presented in Table 6.

**Table 6:** Linear regression model results

Values	B	SE	β	T	p value
Constant	12.039	1.876		6.418	< 0.001
Pre-test PMPs total score	0.538	0.228	0.206	2.364	0.020
Post-test PMPs total score	0.404	0.188	0.188	2.151	0.033
R^2^	0.099				

*B* Estimate of variable’s effect on the model, *SE* Standard Error, β: Standardized coefficients, *PMPs* Patient Management Problems

The backward variable selection method was used. No autocorrelation (Durbin Watson value = 1.808 < 2) or multicollinearity (VIF = 1.082 < 10) were present. The model was found to be significant (F = 7039, *p* = 0.001). Pre-test and post-test PMP scores significantly contributed to the prediction of reflection scores (*p* < 0.05) with one unit change in the pre-test PMP increasing the reflection score 0.538 points and one unit of change in the post-test PMP increasing the reflection score 0.404 points. The equation for predicting reflection scores from pre-test and post-test PMPs was =12.039 + 0.538 (pre-test PMP score) + 0.559 (post-test PMP score).

Opinions and observations of teachers obtained in the program evaluation meeting revealed that the boot camp had increased both the performance and enthusiasm of the students in the following seven-week rotation. Also, the scenario had worked well, and students made genuine efforts to find solutions. In the video debriefing sessions, students appreciated the opportunity of peer learning through inspiration from good performances of role model peers. Students’ approaches were those of novices, and the contextual features of the case had affected the decision-making process and the intervention. Likewise, interns established less contact with forensic or social services than expected. While role-playing, students mostly portrayed the conservative brother as a man of low socioeconomic status. This highlights an important clue to a student prejudice, that conservative men are of low socioeconomic status. Another noteworthy observation was that female students exhibited a more patient and genuine approach toward an interfering companion. It was remarkable that students frequently emphasized that they had learned the empathy needs of the patient, companions, and other team members.

## Discussion

This study describes a one-day drill program using multiple methods of teaching and assessment and aiming to teach clinical decision-making, ethical decision-making, and reflection skills in medical emergency cases, in an integrated approach.

There are several studies in medical education and health professionals’ education literature that integrate either clinical reasoning/decision-making with ethical reasoning/decision-making or reflection skills [13–19]. Thus, the current study seems to be an innovative example for integrating the teaching of clinical reasoning/decision-making, reflection, and ethical reasoning/decision-making.

Despite the fact that integration of clinical skills, ethical decision-making, and reflection skills have emerged as cornerstones of clinical teaching in medical schools recently [13], assessment methods seem to be limited to MCQs in some studies or generic test batteries for ethics [20–24], which put forward the current study in terms of assessing all learning domains integrated. Verbal or written articulation of reflection for assessing reflection skills and rubric scales for analyzing the level of reflection is proposed as appropriate methods [12]. The REFLECT rubric including the levels of Non-reflective: Habitual Action, Non-reflective: Thoughtful Action, Reflective, and Critically Reflective developed by Wald et al. [12] are quite similar to the model employed by the current study’s design.

In this study, a slight but statistically significant difference was detected between pre-test and post-test MCQ scores. In the literature, there are studies on ethics education in medical schools reporting no statistically significant difference [20] or significant increase in post-test scores [21].

The correlation of pre-test and post-test PMP scores with reflection essay scores implied that when students acquire higher levels of cognitive skills, i.e., applying and analyzing, they might perform better in terms of higher-order thinking skills such as reflection. Moreover, the multiple linear regression results support this finding and show that the pre-test and post-test PMP scores contribute significantly to the prediction of reflection scores. These results might support that, better patient management predicts more robust reflective practice.

The spiral model of ethics education in the Acibadem Mehmet Ali Aydinlar University SoM which starts from the first year and introduces more advanced applications or new skills related to those previously covered in the previous years might have contributed to this result. The effect of the contextual features of the case on students’ decision-making and intervention pointed out the need for greater opportunity to work independently and to encounter more complex cases in the previous clinical education. Observing students’ prejudice towards the patient’s brother evoked the need for structured skills training regarding difficult situation management. Besides, fewer students contacted with forensic or social services than expected implied the need to emphasize more when and how to involve forensic and social services in difficult situations.

During this research, we created a toolbox to better capture the richness and diversity of student interactions. A transformative loop, capable of triggering a transformation in self-confidence, supporting the questioning of assumptions, and experiences giving the opportunity to reflect on the actions in both the teachers and the students was formed in the integrated, effective, and supportive learning environment. Students’ situational awareness was improved, and both parties had a view of the traits in the hidden curriculum, such as pre-acceptance of conservative men belonging to low socioeconomic status.

The scarcity of context-specific assessment methods and widespread use of MCQs or generic scales for ethical reasoning in the literature revealed the need for implementing innovative and multi-method assessing ethical reasoning, clinical judgment, and reflection skills in difficult situations [33]. The present study might be regarded as a step forward in that context. Consequently, instead of solely relying on didactic classroom lectures or video demonstration, other environments described in this paper, which enable the students to experience in a kinesthetic fashion, and to reflect on actions of themselves and their groups, could be beneficial. It is thought that similar programs might be of assistance in producing virtuous physicians [31] who also act as moral agents in health care provision.

Future studies in which students reflect on their performances and the use of these writings for following up on their progress might be conducted to assess their reflection skills and self-directed learning.

### Limitations

Students’ reflection essays were not used for following up on their improvement throughout the rotation or the year. No written or structured oral feedback was obtained from the students. Structured qualitative data was not collected.

## Conclusions

This study describes a novel and innovative program utilizing multiple teaching and assessment methods to teach and assess the interrelation between clinical decision-making, ethical decision-making, and reflection with an integrated approach.

Lectures helped students to retrieve previous learning and provided a focus for ethical dilemmas in medical emergency cases. Role-playing the opposite gender and acting as a patient and interfering companion helped the interns to see the gaps in their empathic behaviors and provided a framework for improvement in handling a disorienting dilemma. Debriefing sessions and reflection essays gave them the opportunity for premise reflection, by asking them to reflect on their feelings, thoughts, performances, and their own and peers’ weaknesses or strengths. Reflection essays supplied them with the clues for self-directing their learning and improving their clinical and ethical decision-making skills by formulating action plans to learn further.

## Supplementary Information

**Additional file 1.** Drill scenarios, multiple-choice questions, and patient management problems.

**Additional file 2.**  Research data.

## Data Availability

All data generated or analyzed during this study are included in this published article and its supplementary information files.
