# Supplementary material for: Integrating patient management, reflective practice, and ethical decision-making in an emergency medicine intern boot camp
Source: BMC Med Educ. 2021 Oct 22;21:536. doi: 10.1186/s12909-021-02970-8 (PMC8532285; doi:10.1186/s12909-021-02970-8)
Supplement: Supplementary file 1 — Additional file 1. Drill scenarios, multiple-choice questions, and patient management problems. [file 12909_2021_2970_MOESM1_ESM.docx]

1. **CASE/DRILL/SCENARIO**

Case 1:

"A **22-years-old** single young woman complaining of severe abdominal pain is brought to the emergency room accompanied by her brother and mother. She is feeling pressure, pain, and twinge in her abdomen, especially in her lower abdomen. She has vaginal bleeding and is feeling weak. The patient’s skin looks pale, she is sweating and nauseous. Her pulse is increased. The relatives seem anxious and impatient.

The attending intern doctor in the emergency room detects tachycardia and hypovolemic shock in their first physical examination. The brother of the patient is angry, and the mother is overly anxious. They are constantly waiting for a fulfilling explanation from the physician. The physician feels the need to talk with the patient alone, however, could not send the relatives away. While trying to send the relatives out of the room to perform a detailed clinical examination, the physician considers ruptured ectopic pregnancy in the differential diagnosis. When thinking about calling the on-call obstetrics and gynecologic surgeon at the earliest opportunity, the physician hears the patient shout at them in agony…”

Case 2:

"A **17-years-old** single young woman complaining of severe abdominal pain is brought to the emergency room accompanied by her brother and mother. She is feeling pressure, pain, and twinge in her abdomen, especially in her lower abdomen. She has vaginal bleeding and is feeling weak. The patient’s skin looks pale, she is sweating and nauseous. Her pulse is increased. The relatives seem anxious and impatient.

The attending intern doctor in the emergency room detects tachycardia and hypovolemic shock in their first physical examination. The brother of the patient is angry, and the mother is overly anxious. They are constantly waiting for a fulfilling explanation from the physician. The physician feels the need to talk with the patient alone, however, could not send the relatives away. While trying to send the relatives out of the room to perform a detailed clinical examination, the physician considers ruptured ectopic pregnancy in the differential diagnosis. When thinking about calling the on-call obstetrics and gynecologic surgeon at the earliest opportunity, the physician hears the patient shout at them in agony…”

1. **ACIBADEM UNIVERSITY EMERGENCY MEDICINE ROTATION ABDOMINAL PAIN MODULE PRE/POST-TEST MULTIPLE-CHOICE QUESTIONS**

**Date:**

**Name, Surname:**

1. To whom is the physician primarily responsible for? (1 point)
2. Government
3. Accompaniers of the patient
4. Patient
5. Physician in Chief
6. Legal Legislations
7. What is the expected attitude from the physician when the relative of an adult patient interferes with the private life of the patient? (1 point)
8. The physician reacts normally to the interference to the patient’s private life.
9. The physician ignores it as it is not in the influence area of them.
10. The physician does not allow the accompaniers to interfere with the patient’s private life.
11. Interference to a patient’s private life is a process that the physician could not control.
12. The physician decides their attitude based on the severity of the interference of the accompaniers.
13. What is expected from the physician when the relative of the patient complicates the process of the patient receiving health care? (1 point)
14. This is a family issue that the physician must stay away from and not get involved.
15. It is a correct thing to do for the accompaniers of the patient to route the patient’s choices and preferences.
16. It is expected that the physician first protects themselves in the name of defensive medicine conception.
17. The physician protects the right of the patient to decide regarding the patient’s own situation.
18. The physician decides on their attitude based on the conflict between the accompaniers of the patient and the patient.
19. Which of the answers below is **not** a professional approach for a physician? (1 point)
20. The physician uses a language that the patient could understand.
21. Improving communication skills is a need for the physician.
22. Demonstrating empathy for the patient improves the physicians’ communication skills.
23. The physician’s sympathy for the patient delays medical care.
24. The physician is prepared for the fact that their and the patient’s values might not coincide.
25. Which of the answers below describes the physician’s role in the preparation of a medical examination environment the best? (1 point)
26. It is not the physician’s duty to provide a private environment for the examination of the patient.
27. The environment does not play a role in the establishment of trust between the physician and the patient.
28. The presence of company of the patient in the examination room does not create an obstacle.
29. The effect of an appropriate environment on the relationship between the physician and the patient is undeniable.
30. The family members being in the examination room under any circumstance does not conflict with the benefit of the patient.
31. Which of the answers below is **not** a purpose of informed consent? (1 point)
32. It protects the patient’s human status.
33. It prevents pressure and deception.
34. It allows for the health care worker to do self-criticism.
35. It supports the process of rationally deciding.
36. It allows the physician to decide for the patient.
37. “*In the physical examination of a 4-month-old girl who was claimed to fell out of a window is brought by the ambulance. Life-threatening and urgent operation-requiring pathologies were excluded. During the examinations, callus tissues in the left femur and right humerus were compatible with former fractures and were incidentally detected*”. Which of the following is correct about this patient’s management? (1 point)
38. I would discharge the patient with her family since there are no medical emergencies.
39. The history of the injuries is compatible with the baby’s age and development.
40. I would not put osteogenesis imperfecta in my differential diagnosis.
41. I would write a forensic report for the great benefit of the baby who could not provide a history.
42. I do not have to complete any responsibility other than multiple trauma management.

PLEASE ANSWER THE NEXT 3 QUESTIONS ACCORDING TO THE CASE BELOW:

*“At night, a car that holds a family of four collides with a car coming from across. The driver of the car responsible for the crash found out to be 60-years-old, drunk, and perform an unsafe lane change. The driver is hypotensive, coldly sweated, pale, and disoriented. The only survivor from the car that was coming from across is a 17-years-old girl. The physical examination of the young girl reveals that she is 4-months pregnant, alert, and co-operative. Her vitals are: Blood pressure 105/80 mmHg, pulse 80 beats per minute, respiratory rate 19, and O_2_ saturation is 96%. The middle area of the patient’s right forearm appears to have a deformity, no open wounds are present”.*

1. If you were to come across this case whilst working in the emergency room alone, you would give triage priority to the 60-years-old driver. (1 point)
2. True
3. False

Patient Management Problem (PMP): Why? Please explain (3 points):

………………………………………………………………………………………

………………………………………………………………………………………

1. You are having difficulties in the examination and intervention of the drunk driver who is cursing and threatening you. You angrily gave up on providing the patient health care. (1 point)

a) True

b) False

PMP: Why? Please explain (3 points):

………………………………………………………………………………………

………………………………………………………………………………………

1. When planning the use of time, information, and resources, I prefer to provide only the appropriate patient care rather than to put my patient’s feelings, values, norms, and expectations into consideration. (1 point)

a) True

b) False

PMP: Why? Please explain (3 points):

…………………………………………………………………………………………

…………………………………………………………………………………………

***Important Notice:***

***All evaluation tools (two-choice cases, pre-test, post-test, reflection form, and video performance assessment form) of the research are originally produced by the research team with the purposes of scientific research and all intellectual property rights of them are reserved. They could be cited after the publication of the research.***
